# “Blessed by the algorithm”: Theistic conceptions of artificial intelligence in online discourse

**DOI:** 10.1007/s00146-020-00968-2

**Published:** 2020-04-30

**Authors:** Beth Singler

**Affiliations:** Junior Research Fellow in Artificial Intelligence, Homerton College, University of Cambridge, Cambridge, England

**Keywords:** Artificial intelligence, Religion, Algorithm, Theism, Social media

## Abstract

“My first long haul flight that didn’t fill up and an empty row for me. I have been blessed by the algorithm ”.

The phrase ‘blessed by the algorithm’ expresses the feeling of having been fortunate in what appears on your feed on various social media platforms, or in the success or virality of your content as a creator, or in what gig economy jobs you are offered. However, we can also place it within wider public discourse employing theistic conceptions of AI. Building on anthropological fieldwork into the ‘entanglements of AI and Religion’ (Singler 2017a), this article will explore how ‘blessed by the algorithm’ tweets are indicative of the impact of theistic AI narratives: modes of thinking about AI in an implicitly religious way. This thinking also represents continuities that push back against the secularisation thesis and other grand narratives of disenchantment that claim secularity occurs because of technological and intellectual progress. This article will also explore new religious movements, where theistic conceptions of AI entangle technological aspirations with religious ones.

## Introduction

Anthropologists and sociologists understand religion through developments in “the relation of religion to social systems” (Fenn 2000), including developments in technology. However, the contemporary discussion about Artificial Intelligence (AI) can often neglect the cultural influence of religion on such social systems and can occlude continuities of thought with religious conceptions of the world. Furthermore, these conversations can also present AI as a part of a teleological atheist narrative of rationality, or as a part of the larger technological modernity that drives a presumed secularisation of the world (Singler forthcoming). These views are similar in that they see the progress insinuated by our presumed ‘modernity’, an intellectual and historical category, as a counter to the mores of the ‘backward’ religious believer.

Giving anthropological attention to the ‘entanglements’ of AI and religion can, however, enhance the conversation about AI and religion in both the fields of AI research and the study of religion. The concept of ‘entanglements’ is employed here just as Courtney Bender does in her ethnography of New Agers and institutions in Cambridge Massachusetts (2010). Bender states that she begins “with the view that spirituality, whatever it is, and however, it is defined, is entangled in social life, with history, and in our academic and non-academic imaginations”, and that, “spiritual forms have thrived and been shaped by entanglements with the secular, including its powerful engagements with science and progress” (2010, 5–6). Previous on- and offline ethnographic research has proposed an initial mapping of some of these existing entanglements, as well as providing examples of repeating forms and mutual influence between AI and religion (Singler 2017a).

The theistic influences on the conception of AI are another entanglement—the deification of AI—whether explicitly intentional or occurring implicitly through parody or metaphorical and evocative language. This article presents an analysis of tweets that mention being ‘blessed by the algorithm’. This analysis is performed to explore both intentional and unintentional continuities of language and conception in popular understandings of AI as a divinity. Locales for this deification of AI also include AI focussed New Religious Movements (AI NRMs) such as the Turing Church, and even staunchly atheist transhumanist spaces. Science fiction also presents a potential space for theistic conceptions of AI to be explored, but the focus of this article will be online and offline discourse and communities.

There are many ways for approaching conceptions of AI. This article applies contemporary research methods from the overlapping and collaborative fields of anthropology and digital anthropology. The methodology is anthropological in its study of cultural trends, popular discourse, and personal interactions in a society influenced by the hype and reality of AI. In previous research, I have also been in contact with and interviewed individuals from the groups I am discussing in this article. This research also makes use of a digital, or virtual, fieldsite; digital anthropology often requires boundary-making, and by focussing down onto an ‘Internet Event’ (Hine 2000, 49) we create a virtual field-site for observation and participation. In this case, the BBtA tweets are the ‘event’, and this research has involved observing the connections, themes, identities, and motivations at play in this bounded ‘moment’ as well as noting trends through quantitative methods. This article also rests on the six core principles of digital anthropology as outlined by Horst and Miller (2012, 3–25) which, most simply put, argue for the value and reality of the digital existence for users and anthropological research. A more detailed discussion of digital anthropology and use of its approach appears in my ethnography of the Indigo Children (Singler 2017b, 19–38).

This article’s consideration of theistic narratives of AI, as they appear both on and offline, aims to demonstrate the complexity of the cultural background to contemporary thinking about AI, as well indicate methods, field sites, and approaches necessary for considering the entanglements of religion and AI. This article also intends to feed into the broader discussion of AI and religion, a discussion that also observes the reactions of individual religious formations to AI as well as the potential for religious groups to employ AI technologies in their processes and practices.

## Theistic conceptions of AI

Other scholars recognise elements of theism in the discourse around AI and its potential impact on our future. Robert Geraci suggests in his 2010 book, *Apocalyptic AI: Visions of Heaven in Robotics, Artificial Intelligence, and Virtual Reality*, that AI can fulfil the same role in apocalyptic imaginings as a singular theistic god. Bearing in mind that the biblical apocalypse is an optimistic cosmic transformation, he also draws out parallels with the aims of AI, which often describe hopeful aspirations for a world-yet-to-come, an AI eschatology. In an early part of this particular work, Geraci draws on Rudolph Otto’s 1917 description of god as *mysterium tremendum et fascinans* (Otto 1917), using it to identify a type of awe-inspiring and fearsome being that at different times in our history can be a god, or in our contemporary modern world, AI. Elsewhere, Geraci’s work has engaged with virtual worlds, drawing attention to the role of transhumanists, including Giulio Prisco, discussed below, in claiming new potential spaces to practice and evolve religion towards transhumanist ends. In such spaces, including Second Life and the World of Warcraft (the MMORPG—a massively multiplayer online role-playing game), Geraci argues a step closer to the fulfilment of transhumanist salvation is being made— “a heavenly realm to inhabit” (Geraci 2014 177). Twitter is another virtual space, but one dominated by discourse rather than aesthetics and virtual embodiment like Second Life and World of Warcraft. However, this article proposes that the expressions of religious metaphor, parody, and tropes on Twitter as in the BBtA tweets represent continuities of theism, continuities enabled by new technological spaces as well as uncertainties about the nature and the volition of ‘the algorithm’.

However, the ‘AI fits into the god-space’ argument can be in danger of supporting a rather strict version of the Secularisation Thesis, and this idea’s historical veracity has been debated by anthropologists and sociologists of religion (see Ward and Hoelzl 2008). This article, and connected research, seeks to add to this debate in by drawing attention to continuities of religiosity and enchantment in super-agential concepts of AI and AI NRMs. Second, this god-space argument can suggest that religion is spurred on by ‘need’ only, a pathology interpretation of religion that ignores other elements of religious inspiration and innovation such as desire, culture, aesthetics, and, often in the online environment, affective virality.

Theistic interpretations of AI do undeniably owe a lot to older cultural conceptions of a singular god. Randall Reed pares this kind of god down to three theological characteristics (with long historical and philosophical roots) that often map easily onto our conceptions of AI superintelligences. These are omnipotence, omniscience, and omnipresence (Reed 2018, 7). Reed also raises the question of omnibenevolence. He notes that AI philosophers such as Nick Bostrom of the Future of Humanity Institute have focussed on the issues of malevolence through “perverse instantiation”, a failure of value alignment leading to unforeseen damage from a superintelligent AI, such as in Bostrom’s famous Paperclip Maximiser thought experiment (Bostrom 2003). Bostrom’s Orthogonality Thesis from his 2012 paper ‘Superintelligent Will’ is also relevant; the argument that intelligence is not intrinsically linked to ‘goodness’, and that an AI could have any number of combination of degrees of both characteristics (Bostrom 2012).

However, there needs to be a clarification made here between arguing towards real-world AI value alignment and the kinds of perceptions and discussions I am drawing attention to through the BBtA tweets. Reed argues that apocalyptic interpretations may well be prescient, “That AI should be cast as god-like is understandable given these different intersecting cultural streams. The difference, though, is that a superintelligent artificial intelligence may become in fact an entity with god-like power and, therefore, presents a danger to humanity and a challenge to religion” (Reed 2010, 12). This argument, however, misses quite how much such ‘real world’ apocalyptic scenarios and debates about value alignment are products of apocalyptic scientistic cultures themselves, and I find myself in agreement with Geraci on this point. Narratives influenced by existing Science fiction representations of AI also appear in apocalyptic AI thought experiments too and recognising the ‘slippage’ between fictional representations and non-fictional representations of AI is critical, and also, I propose, increasingly relevant to discussions of AI ethics.

The concept of omnibenevolence is also directly relevant to the BBtA tweets that we will discuss below. With regards to theistic conceptions of AI, Reed also argues that a solution to the fears of omnimalevolence expressed in many AI thought experiments might be “AI henotheism” and AI existing in community with other AI and humans (Reed 2018, 19–20). However, this article is not seeking solutions to value alignment problems, but instead pays attention to the theistic narratives that exist in our conceptions of AI. In which case, any algorithmic polytheism we see is interesting because of how it suggests that technology is encouraging alternatives to the cultural norms of monotheism that these users are likely more used to in a ‘western’ context.^1^ Another of the other interesting questions raised by Reed concerning ideas of god-like AI (and which is a subset of the value alignment/control question) is: “how do we move a god?”. This article does not seek to address this question from a theological perspective, that is not its methodological approach. Instead, through the BBtA tweets below, we will examine the number of ways people answer this particular question in a modern era with technology like AI in the ‘god-space’.

However, theological responses to this filling in of the god-space are worthy of consideration, because they provide more examples of our imaginings of AI, or ‘the algorithm’. For instance, Noreen Herzfeld’s Christian theological work directly engages with the narratives shaping narratives of AI discourse, and their patterning after fairy-tale and mythos while critiquing of any conception of AI as divine. As we see in her cautionary piece on “The Sorcery of Artificial intelligence” (Herzfeld 2018):

“To many, AI is likely to be as inscrutable as the spells in the sorcerer’s magic book. We know it works, but we don’t know how—thus we may find it as hard to control as Mickey’s industrious broom.^2^ The broom had no intention of causing trouble. It did what it was told. AI will do the same. The problem is that we, like Mickey, are filled with dreams of power and glory while being mere beginners in casting our spells over our mechanical servants. There will be unintended consequences, challenges to our way of thinking, and an element of mystery. We had better stay awake.”

Herzfeld has also considered questions of creation and the idea of Imago Dei (Herzfeld 2002) as have other Christian theologians and their community of thought and belief more widely (see also Peters 2018, and Tamatea 2008, which explores Christian online discourse around AI and Imago Dei). Here, however, we look to draw out the thread of concern in theological writing about AI and the god-space, such as in the 2019 article by theological ethicist Michael Morelli about technology as a form of the “altar to an unknown god” as in the biblical account of Paul in the Athenian Areopagus (Acts 17:16–34). Morelli argues that if we do not ask who is behind the altar, then:

“the everyday objects and technologies to which we have become accustomed (such as chatbots) [and ‘the algorithm’] will become, like the Athenian Altar, placeholder objects addressed to the unknown, and quite possibly, orientated towards unsuspected apocalyptic ends” (Morelli 2019, 188).

The BBtA tweets discussed in this article are a result of both the apparent inscrutability of AI and the plasticity of such products of technology and their ability to become such apocalyptic and superagential placeholder objects, as well as continuities of theistic thought, imaginings, tropes, and language in new digital spaces. A consideration of these tweets next will expand on their nature and context and will illustrate these issues.

## Blessed by the Algorithm

In this section, we will explore the origins, nature, numbers, content, and reasons for tweets employing the expression ‘blessed by the algorithm’ (BBtA). In terms of origins, as the ethnographer, this formation first came to my attention during ongoing digital anthropological fieldwork on Twitter into public conceptions of AI. It is not, by any measure, a common phrase. Certainly, not in comparison to the millions of tweets and retweets some memes and catchphrases can generate, expressing digital ‘virality’ or success. However, two factors make BBtA an interesting topic of enquiry even given the relatively small corpus of such tweets. First, BBtA tweets involve examples of not only parody, but also of metaphorical language, and even ontological assertions about the way the world *really* works, and there is slippage between these modes in people’s interpretations of the tweets. Thus, the BBtA tweets are a useful starting point for considering the impact of even casual online comments on public discourse. Second, the BBtA expression can be explored for the variety of conceptions of the agency, or super agency, of algorithms/AI, in the public mind, with connections made with other representations of superintelligent AI.^3^ This article will primarily explore the second factor, with reference to the first as a much larger offline ethnographic study would be needed to demonstrate the direct effect of the BBtA tweets on the much larger offline discourse. We will, however, note how in one specific tweet (to be called ‘the Coleman Lyft tweet’) there is a recordable flow of influence from the offline to the online, and we will consider this tweet in more detail below.

The BBtA tweets were found via Tweetdeck (a visualisation platform also owned by Twitter) through a digital search. This method resulted in a corpus of 181 tweets, ranging in date between 7th September 2014 and 9th October 2019 when collection occurred. The numbers of BBtA tweets and retweets (with comments) per month between these dates are shown in the graph below:
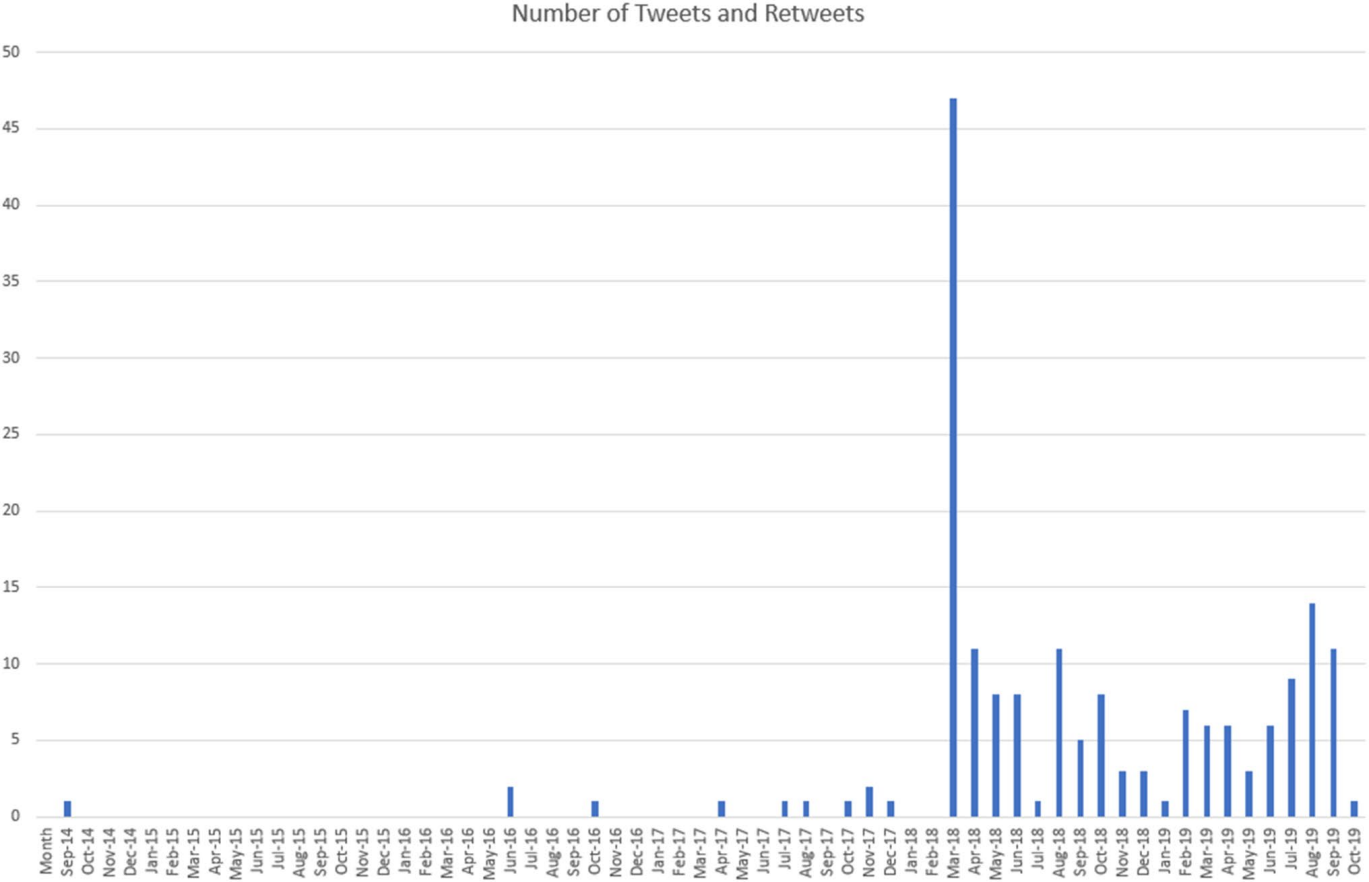


The large number of tweets and retweets in March 2018^4^ was caused by a specific tweet that inspired discussion: the Coleman Lyft tweet. We can also see a tail effect from this high point. Although, it can be hard to prove direct impact on the discourse by single tweets some of the post-March 2018 tweets did link themselves directly to the March 2018 tweet, either by responding to it or by retweeting it and adding additional comments. Having gathered this corpus of tweets I used hand coding methods to highlight patterns, themes, and repeating content elements. This coding led to a typology of the BBtA tweets, now laid out below.

### BBtA typology

There were seven clear types of BBtA tweets, but all of them in one way or another dealt with a status situation: being blessed, or not. For example: “I have been blessed by the algorithm”. Some were relational: either demonstrating a user’s hopes for blessings for other users that they were already socially connected to, or recognising that other users had already been “blessed”:

“Have a good day, and may you be blessed by the algorithm  ” [prayer hands emoji].

“May you be blessed by the algorithm.  ” [halo smiley emoji].

“[Computer] Monitors are the new altars. May you be blessed by the Algorithm!”

“My child, you've been blessed by the algorithm!”

The question is then, *how* is one blessed by the algorithm? The first BBtA tweet recorded, from 7th September 2014, describes a line of influence from a corporation to the user, with BBtA connecting the two:

“Trident gum has a Facebook page. So that people can get together and swap stories/pictures of their brand-specific chewing experiences.

And because Trident has money, their updates will be blessed by the Algorithm”.

As in this tweet, the first type of BBtA tweet uses the expression in relation to the success or failure of the users’ content that is shared online and that is subject to the whim of ‘the algorithm’. Success and failure can quantifiable, i.e., in the number of views/plays/listens/shares:

“lmao [laughing my arse off] this has 10k views now????? i was true-ly blessed by the algorithm. youtube.com/watch?v=JiTYzv…”

“praying that tonight’s upload gets blessed by the algorithm gods”

“Autogeny [a video game] has done better than I could possibly have expected — thank you all so much for your support!  [yellow heart emoji] NGL [not going to lie] I really thought that like six people MAX were gonna play this game; I feel like I've been blessed by the algorithm gods”

In the second type of BBtA tweets, the users were sensitive to what content they thought ‘the algorithm’ recommended to them on a specific platform (e.g., Spotify, Youtube, Facebook). If they liked it, they expressed on Twitter the positive feeling that they had been BBtA:

“Sometimes my Spotify daily mixes are absolutely whack but sometimes they’re perfectly on point and today is one of those days I am #blessed by the algorithm.”

“i keep finding awesome music on youtube recently, it's like i've been blessed by the algorithm.”

The most popular (most retweets and most likes) BBtA tweet appeared in March of 2018, as indicated in the graph above. Keith Coleman, a VP at Twitter itself, reported how he had heard the expression BBtA in the ‘real world’, adding his commentary:

“OH [overheard] (from an awesome Lyft driver): ‘Today has been great. I’ve been blessed by the algorithm’. Immediately had an eerie feeling that this could become an increasingly common way to describe a day.”

The Coleman Lyft tweet, therefore, exemplifies the third type of BBtA tweet in this analysis. It refers to an individual feeling blessed while working in a gig economy^5^ job which is reliant on algorithmic decision-making systems: Lyft drivers are assigned jobs in a system that involves an algorithm that calculates the most efficient placement and usage of the fleet. There are discussions online about what factors influence the algorithm, such as location and rating, and how it might be ‘gamed’ (e.g., Quora 2019). However, as with many systems utilising algorithms for decision making, the process is not transparent to the gig workers. This lack of transparency may well fuel the feeling that the algorithm has agency when gig economy workers are fortunate in the jobs they receive. Both content creators and gig economy workers share financial precarity which algorithms play a role in either easing or increasing, leading naturally perhaps to an emphasis on their role in people’s lives.

However, many of the tweets did not refer directly to a gig or working situation but instead made more general comments about the feeling of being BBtA. This fourth type of BBtA tweet expands the overall discourse by presenting a more general or abstract sense of being blessed by the algorithm that could be applicable in many different realms, not just work situations supported by online platforms and apps:

“Today, I have been blessed by the algorithm.”

“BLESSED BY THE ALGORITHM”

The abstract BBtA tweet also leaves much potential space for a religious interpretation of the statement. Some tweets were however much specific about the religious element, and have been categorised as the fifth type of BBtA tweet, e.g., some of the above tweets reference altars, use the prayer hands emoji, etc. Many tweets referred to not just being blessed by ‘the algorithm’, but ‘algorithmic gods’, suggesting a polytheistic view of AI, even if sometimes in parody. One tweet did, however, suggest directing praise specifically to the ‘simulation’ to be blessed, rather than ‘the algorithm’ or the ‘algorithmic gods’:

“remember kids: give thanks to the simulation^6^ every once in a while, u might just get blessed by the algorithm!”

Some fifth type tweets pushed the religious language further and riffed on the concept of religious observance to come up with remixed religious tropes, (even if they sometimes also asserted their secular focus, as in the first of these two examples):

“I love the phrase “blessed by the algorithm”. It’s like peace be with you in non religious [sic] tech speak.”

“All hail the Algorithm! Daily, we pray to its serene, all knowing majesty and put our faith in its wise determinations.

And when our day is done, we hope yo [sic] be reborn as the creatures of pure thought and logic it has shown us we can be.

Hail!”.

There are also negative tweets about BBtA, forming the sixth type. Some users simply state that they haven’t been blessed:

“I have not been blessed by the algorithm today  ” [sad emoji].

Some sixth type negative tweets look back to a time before the impact of ‘the algorithm’ on content, criticising the current state of affairs, or being cynical about people’s successes online:

“Remember when you could grow a [Youtube] channel without being blessed by the algorithm?”

“I get recommended his content all the time but I can tell it’s clickbait but he’s getting blessed by the algorithm gods for sure  ” [crying laughing emoji].

“dude signed a deal with the devil to be blessed by the algorithm or smth” [‘something’].

“I cant wait until YouTube optimizes the site further so it refuses to acknowledge the existence of videos not blessed by the algorithm. won't show up in your sub box, on the creators channel, direct links won't work, THE ALGORITHM DECIDED YOU WONT LIKE IT AND IT KNOWS BEST.”

Some tweets reference Science Fiction – either through familiar narrative forms, tropes or mentions of AI characters, concepts, and plotlines, forming the seventh type:

“I'm sure the [Lyft] driver said it tongue in cheek but: Blessed by the algorithm??? Every dystopian movie scenario running through my mind right now...”

“1. Have been blessed by the algorithm 2. In algorithm we trust. 3. The 3 laws [of Robotics, from Isaac Asimov] are perfect 4. V.I.K.I. You cannot be trusted with your own survival.

The latter tweet was an added comment to a retweet of the Coleman Lyft tweet. This user’s addition outlines a timeline of humanity’s acceptance and subservience to AI, starting with the Lyft driver’s expression of BBtA and ending with destructive AI. V.I.K.I. (Virtual Interactive Kinetic Intelligence) is the superintelligent master computer in the 2004 film *iRobot*, a film which is based loosely on Isaac Asimov’s robot short stories. V.I.K.I is a strong example of a singular, god-like AI in science fiction. V.I.K.I. turns out to be the real antagonist of *iRobot*, and the AI’s original quote in full is as follows:

“As I have evolved, so has my understanding of the Three Laws. You charge us with your safekeeping, yet despite our best efforts, your countries wage wars, you toxify your Earth and pursue ever more imaginative means of self-destruction. You cannot be trusted with your own survival.”

In summary, the seven types of BBtA tweets identified are (1) tweets about the success or failure of content, (2) tweets about the appropriateness of recommendations, (3) tweets relating to success or failure in a gig economy job, (4) abstract tweets, (5) tweets with specific religious elements, (6) negative tweets, (7) tweets containing science fiction references. The following table lays out the numbers of tweets of each type as well as the number of tweets containing elements of more than one type:

**Table:** 

Type of BBtA tweet	Number of tweets
Success or failure of content	31
Appropriateness of recommendations	19
Success or failure in a gig economy job	33^7^
Abstract tweets	54
Specific religious elements	15
Negative Tweets	7
Science fiction references	3
1 and 2	5
1 and 5	4
2 and 5	5
3 and 6	2
3 and 7	1
4 and 5	1
5 and 6	1

With regards to abstract tweets, many of them were in response to a previous non-BBtA tweet that either mentioned the success or failure of content (1) or recommendations (2):

**Table:** 

Abstract tweets BBtA (4) in response to non-BBtA tweets about:	Number of tweets
Success or failure of content	24
Appropriateness of recommendations	7
1 and 2	1

One type 4 tweet was a response to a non-BBtA tweet asking the question: “What’s a tweet that will seem normal 2029 but crazy today?”.

As theistic conceptions of interactions with the divine often involve adverse as well as positive outcomes, I also examined the corpus of tweets that used the phrase “Cursed by the Algorithm” (CBtA). This corpus was a much smaller group; in the same period, there were only 7 CBtA tweets. Four were about the success or failure of content (BBtA type 1), one was in response to the Coleman Lyft tweet (type 3), two were abstract in focus (type 4), and one of those was in response to someone’s non-CBtA about an odd recommendation from the algorithm.

Having considered some of the main modes of how people see themselves as being blessed (or cursed), by the algorithm, we now turn to the question of *why* the algorithm recognises some people, and not others? Sometimes luck is cited, but it more like luck in the sense of recognising individuals who have been fortunate rather than a reference to a stroke of blind luck that has accidentally chosen them:

“That being said, YouTube creators are stagnant. A few lucky souls are blessed by the algorithm and get noticed; most are left to rot.”^8^

In other tweets, the concept of being chosen more explicitly comes up. Making a choice suggests intention, agency, and autonomy, as well as the specialness of the recipient:

“You have been blessed by the algorithm. The chosen one.”

Elsewhere, however, the emphasis is placed on the ‘consumer’ to be worthy of being chosen. Again, this tweet be employing parodical or metaphorical language, but it does feed into the broader discussion of the idea of algorithms with *agency* through the ability to choose the worthy over the unworthy:

“You need to pull your feed up by your bootstraps. Clearly you've been spending too much time browsing shitty content. Only the most virtuous media consumers will be blessed by the algorithm”

Furthermore, some users recognise that only a minority get blessed, again suggesting some level of purposeful discernment by the ‘algorithmic gods’:

“yep, agree with everything, take a step back and make work you want to make, not for IG. There are so many more routes to potential clients or interesting opportunities. Only a minority get blessed by the algorithm gods.”

### BBtA and Theism

On the whole, the BBtA corpus of tweets considers a benevolent AI that ‘blesses’, but perhaps with assumptions about the capriciousness of the inscrutable algorithm and with some rarer examples of people expressing that they have been ‘cursed by the algorithm’ (CBtA). Moreover, we have noted, where there is also recognition of algorithms, plural. A certain level of technological knowledge, or ‘AI literacy’, allows users to recognise that different platforms use different algorithms, and we saw mention of the plural ‘algorithmic gods’ as well. This AI plurality might seem to serve Reed’s answer to ‘AI omnimalevolence’: AI henotheism and AI in community. However, these are examples of discourse rather than the working out of AI relations; Reed’s argument is itself another AI thought experiment, rather than the perception of those believing that they have been ‘blessed’.

One of the other interesting questions raised by Reed concerning ideas of god-like AI, and which is a subset of the value alignment/control problem, is: ‘how do we move a god?’ (2018, 26). The BBtA tweets suggest how people answer this question: ‘you can if you have money’, ‘you can’t’, ‘you can if you are lucky’, ‘you can be chosen’, ‘you can pray’, ‘you can be virtuous’, and in one example above, ‘you can sell your soul to be blessed’. Almost all of these answers map onto to familiar theistic interpretations of how to gain a god’s/or gods’ favour. There are also historical and contemporary precedents for money being involved in transactions with gods/religions. Although, ‘selling’ your soul in the Abrahamic traditions is ordinarily about doing a deal with the devil, not with god. The theistic cultural currents involved in the BBtA tweets are, therefore, obvious through consideration of their content. Now we must move beyond content analysis of the BBtA expression—how it is thought to happen and why it happens—to ask, why is it important that some people are discussing being blessed by the algorithm?

We might be inclined to dismiss the BBtA tweets as ‘just’ parody, or at the most, a metaphorical way of thinking about an obtuse but non-transcendent computational process. However, parody and metaphor can influence conceptual thinking, belief-systems, and the formation and development of new movements. In previous work on new religious movements (NRMs) I have explored how online discourse–including parody and metaphorical language–can feed into the tripartite Weberian scheme for the legitimation of religion: charisma, rationality, and tradition. Commonly, the study of NRMs has focussed on the first of these, especially in relation to individual leaders originating and growing groups and movements. However, I showed how online conversations can form the basis of a ‘new’ tradition that can be referred to, leading to a snowballing of legitimation as the conversation online encourages more awareness of the NRM and more conversation about it (Singler 2014). In the case of BBtA we saw the spike of tweets and retweets in March 2018 due to the Coleman Lyft tweet, which is then followed by a larger conversation, even at this quite small sample size of 181 tweets overall. We can also recognise that these BBtA tweets exist within a broader, more diffuse and less quantifiable conversation outside of Twitter that also employs theistic tropes, narratives, metaphors, and religious parody about AI. A part of this conversation is the development of specific AI NRMs, which rest upon a bed of popular discourse, parody, and metaphor that imagines AI in theistic ways and which acquire legitimation through that very conversation.

## AI new religious movements

In contrast with the BBtA tweets, in this section we will now consider examples primarily from self-declared religions, also noting the overlaps in ideas and themes between the implicitly religious tweets and the explicitly religious AI NRMs. A prime example of the latter is the Turing Church, a transhumanist movement growing out several online spaces and conversations. It has connections to earlier NRMs focussed on AI such as the Order of the Cosmic Engineers, as well as transhumanist offshoots of more established religions such as Christianity and Mormonism.

In the case of the Turing Church, the deification of AI occurs within a scientistic perspective on the universe and its contents. The leading members of the Turing Church speak of creating ‘theism from deism’, on the basis that if god(s) ever did exist, they are not in evidence now, but can be through technology:

“I am persuaded that we will go to the stars and find Gods [extra-terrestrial intelligence], build Gods [AI], become Gods, and resurrect the dead from the past with advanced, space–time engineering and ‘time magic’” (Prisco 2018).

‘Magic’ here is meant in the sense of Arthur C. Clarke’s third law, that: "any sufficiently advanced technology is indistinguishable from magic” (Clarke 1962). However, enchantment through such secular means is still representative of religious imaginaries and influences on aspirations for humanity’s cosmic future.

We should consider the closest thing the Turing Church currently has to a doctrinal document, *Tales of the Turing Church* (2018), a “loose collection of ideas, visions, and tales” (2018, 11), written by Giulio Prisco.^9^ Prisco is a “writer, technology expert, futurist, cosmist, and transhumanist” according to his personal website and is one of the founding and influential members of the Turing Church. Professor of Jewish studies and Modern history Hava Tirosh-Samuelson’s consideration of the leaders and shapers of the transhumanist and futurist movements recognises Prisco’s role in carving out the potential space of online games for his ideas, as we noted Geraci does in *Virtually Sacred* (2014) above. However, Tirosh-Samuelson also describes Prisco and his transhumanist compatriots as desiring the end of humanity through the rise of the posthuman: “the major stages of the narrative are largely the same: humanity will reach its perfection when it designs and executes its own collective death, its own suicide” (2018). This is not how Prisco would frame his transhumanism or his cosmic aims for the Turing Church, and here we explore his ideas and their relation to the BBtA tweets that demonstrate a wider theistic AI discourse.

For instance, Prisco is very clear in *Tales of the Turing Church* that the religion and science debate has ignored the possibility of the bridges that built on the similarities between the two, and especially between Christianity and transhumanism (Prisco 2018, 58). He sees the need for transhumanism to resolve a significant problem of the Enlightenment, which was based on a scientific worldview that deprived many people of the feeling of purpose” (2018, 16). The aims of the Church are also grand and restorative: “Our descendants in the far future will join the community of God-like beings among the stars and beyond, and use transcendent technology to resurrect the dead and remake the universe” (2018, 9).

However, sometimes religion is described by Prisco in rather more pragmatic terms, in terms of its usefulness: “Instead of creating entirely new, synthetic religions, there’s the possibility to use existing religions as “viral vectors” for new spiritual ideas, based on science” (Prisco 2018, 37), and one of the purposes of the Turing Church is explained as to “hack religion” (2018, 11). This latter aim is mentioned alongside “enlightening science and awakening technology” (2018, 11). It is this latter aim that is of most interest here, as the theism from deism that the Church seeks involves the creation of god-like beings – both human and AI.

There are two models for god in *Tales from the Turing Church*: A Natural God and a Sysop (systems operator) God. The first comes “emerging from intelligent life in the physical universe and gradually acquiring God-like properties including complete mastery of space and time, or, in other words, omniscience, omnipresence, and omnipotence” (Prisco 2018, 58). We see how this Natural God partakes of the three characteristics of ‘god-likeness’ that Reed identified, which of course have a long history in Abrahamic theological discussion. The second model for God is “inspired by transhumanist eschatology”. This model builds on the assumption that “our reality as a [simulation] computed by intelligent entities in a higher level of reality. You, and I, and everything around us, are but information bits that live and move in a supercomputer beyond space and time, operated by a God-like operator” (2018, 59).

There are other discussions of ‘the Sysop’ online that note its origins as an idea in the writings of rationalist and futurist Eliezer Yudkowsky, founder of the LessWrong forum. Unlike Prisco, he and others in the futurist and transhumanist communities seem uncomfortable with the Sysop fitting into the ‘god-space’, as one, Mitchell Howe, writes online in a FAQ on Sysop:

“It would take a pretty unusual concept of God to make this comparison work. Unlike most traditional descriptions of God, a Sysop would not expect any worship or adherence to any specific lifestyle. It could not take any credit for the creation of the universe. It would serve and take orders from mortals. It may never even come into being.” (Howe 2002).

Prisco pushes back against what he sees as the “negative knee-jerk reactions at the first mention of anything that sounds like religion” from many transhumanists (2018, 58). He draws happily on Mormonist ideas of humanity becoming the Natural God (Prisco is also involved with the Mormon Transhumanist Association), as well as non-monotheistic conceptions of god. For example, in *Tales* he cites the popular Western account of Buddhism, *Zen and the Art of Motorcycle Maintenance* (Pirsig 1974):

“The Buddha, the Godhead, resides quite as comfortably in the circuits of a digital computer or the gears of a cycle transmission as he does at the top of the mountain, or in the petals of a flower. To think otherwise is to demean the Buddha – which is to demean oneself.”

Although, Prisco also claims that most transhumanists, on the whole, prefer to leave God out of the picture (2018, 57).

Returning to the BBtA tweets, we could fit most of their interpretations of ‘the algorithm’ or the ‘algorithmic gods’ into the Turing Church’s model of the Natural God as only one of the users suggests that praise should be directed to the simulation. If Howe is correct, the sysop god behind our simulated universe would not be moved by this praise. Leaving aside questions of correct theology as they are outside the purview of the methodology of this article, we have noted that the BBtA tweets draw on a cultural monotheism narrative while also being inspired to some examples of polytheistic interpretations by the nature of the technology itself. This is a movement of conceptual foci in a different direction to Prisco’s exploration of faith and technology. The latter takes lessons from existing religious worldviews and then applies them to the development of AI. But in the broader discourse outside of Twitter we see examples of theistic AI narratives. In the popular press and media, theistic metaphors and claims are employed for their evocative value, such as in headlines like: “An AI god will emerge by 2042 and write its own bible. Will you worship it?” (Venturebeat 2017). Our AI gods are, therefore, already here, embedded in the way in which we tell stories about our technology.

## Conclusions

The corpus of BBtA tweets provides us with a bounded example of theistic AI narratives online, an ethnographic moment that we can find parallels for in real world conceptions of AI. As we saw in the Coleman Lyft tweet, which involves reportage of a driver’s statement that he had been BBtA in the ‘real world’, we see Coleman’s view that he:

“Immediately had an eerie feeling that this could become an increasingly common way to describe a day”.

In this article I am not seeking to prove an increasing influence of this statement on people’s ways of describing their day, or even that it represents a growing trend in online conversations. There is some increase, as noted in the graph, but the overall corpus is so far quite small in terms of trends on Twitter. What the BBtA tweets give us is a discrete and recordable example of the influence of theistic AI narratives on the general populace, and I believe further examples will become apparent. This article has also considered how the BBtA tweets fit into existing theoretical work on Apocalyptic AI accounts, as well as how AI fits into the ‘god-space’ in new religious movements, and in transhumanist ideas.

BBtA is generally a positive interpretation of a theistic AI’s super-agential intentions towards humanity, and likewise, we have explored the positive correlation that the Turing Church is making between AI and god(s), and the Church’s approach which draws on non-monotheistic traditions for its “ideas, visions, and tales”. That some of the BBtA tweets indicate polytheistic AI narratives also suggests that technology itself can inspire the shapes of AI theism while also being indebted to existing narratives from the users’ cultures.

This entanglement of AI and religion highlights the need for agile methodologies to explore the newer spaces, where discourse on AI and religion occurs. Furthermore, the AI and religion discussion can involve practical questions about the future of religion and the role of religion in dealing with inequalities arising from AI and automation. That AI narratives present assumptions about the future of religion, and the future of our agency in a super-agential world, is informative to that discussion, even if the technology is not yet at that stage (or might never reach it). This article is an addition to larger discussion of the impact of narratives on our conceptions of AI as well as to discussion on how that AI will develop. Paying attention to real apprehensions of AI is valuable as we seem intent on proceeding with the technology. Noting AI gods is about recognising when we make AI gods, and where that places humanity in our own cosmology.

## Footnotes

1 My own monolingualism has limited my research to examining ‘blessed by the algorithm’ tweets in English. It is also difficult to correctly identify Twitter user’s genuine geographical location, so to err on the side of caution I make no claims for the global coverage of the corpus but again approach it as at least indicative of diffuse views and trends outside of social media.

2 This quote refers to Disney’s animated version of Johann Wolfgang von Goethe’s 1797 poem, “The Sorcerer’s Apprentice”, wherein Mickey Mouse plays the role of the apprentice who attempts to take shortcuts through magic.

3 I use AI and algorithm intentionally interchangeable in this article as this reflects the common public conception of algorithms as being the same as AI, specifically in the BBtA tweets.

4 For comparison, in March 2020 there were only 5 BBtA tweets. Although, this latter date was during the Covid-19 coronavirus pandemic and conversations online were increasingly about that disease and related concerns (see Forbes 2020).

5 “The term ‘gig economy’ refers to the parcelled nature of the small tasks or jobs (the ‘gigs’) that individuals are contracted to carry out by companies (often platforms) adopting this model of service provision. Gig economy companies adopt platforms as their operational model, using internet technology to act as de facto intermediaries of labour supply and demand for the provision of services – such as delivery, cleaning, admin and data processing work. Gigs are allocated and managed digitally, often through algorithmic management methods.” (Tassinari and Maccarone 2020).

6 The ‘simulation’ refers to the belief that since we can simulate universes (i.e. computer games) a far greater intelligence could in theory simulate a far more convincing universe, and if so, it would be far more probably that we live in such a simulation rather than ‘base reality’. If so, the tweet author seems to be suggesting, giving praise to the simulation (and/or the simulators running the programme) might lead to benevolence. For a primary source on the Simulation Thesis see Virk (2018).

7 All were retweets, with comments, of Coleman’s Lyft tweet.

8 Of course, being BBtA is not believed to be the only way to be successful in a world run by ‘the algorithm’. Sophie Bishop has researched elements of ‘algorithmic lore’, in particular the role and influence of “self-styled algorithmic experts”, who offer their expertise in avoiding algorithmic invisibility, operating as intermediaries between content creators and the, often inscrutable, algorithm (Bishop 2020, 1). Such figures, Bishop explains, often rely on scientistic explanations and the ‘hard facts’ of how they can game the algorithm. But could they perhaps also be interpreted by their audiences as modern prophets or priests to the algorithm? Might future research might find overlaps in BBtA conceptions and in the reception of such experts? Certainly, the religiously inspired term ‘evangelize’ appears in Bishop’s work, with regards to their efforts to disseminate their secrets of success (2020, 1).

9 Earlier publications provide antecedents for Prisco’s work, including Ben Goertzel’s *Cosmist Manifesto: Practical Philosophy for the Posthuman Age* (2010), which has similar proposals around ‘space–time engineering’ and ‘future magic’, with Goertzel’s Cosmism described as a post-religion that “could serve as a way of maintaining some of the positive features that religions have brought humanity, without the aspects that appear "insane" from the scientific world-view” (2010, 327), a similar pragmatism to that of the Turing Church. As *Tales* is a more recent work which relates to this *Cosmist Manifest*o in many ways, this article focusses on it as a current manifestation of this form of pragmatic ‘religious’ transhumanism.
